# Renal and multisystem effectiveness of 3.9 years of migalastat in a global real‐world cohort: Results from the followME Fabry Pathfinders registry

**DOI:** 10.1002/jimd.12771

**Published:** 2024-07-19

**Authors:** Derralynn A. Hughes, Gere Sunder‐Plassmann, Ana Jovanovic, Eva Brand, Michael L. West, Daniel G. Bichet, Antonio Pisani, Albina Nowak, Roser Torra, Aneal Khan, Olga Azevedo, Anna Lehman, Aleš Linhart, Jasmine Rutecki, Joseph D. Giuliano, Eva Krusinska, Peter Nordbeck

**Affiliations:** ^1^ Lysosomal Storage Disorders Unit Royal Free London NHS Foundation Trust and University College London London UK; ^2^ Division of Nephrology and Dialysis, Department of Medicine III Medical University of Vienna Vienna Austria; ^3^ Northern Care Alliance NHS Foundation Trust Salford UK; ^4^ Internal Medicine D, Department of Nephrology, Hypertension and Rheumatology, Interdisciplinary Fabry Center Münster University Hospital Münster Münster Germany; ^5^ Department of Medicine Dalhousie University Halifax Nova Scotia Canada; ^6^ Department of Medicine, Hôpital du Sacré‐Coeur University of Montréal Montréal Quebec Canada; ^7^ Department of Public Health, Nephrology Unit Federico II University Hospital Naples Italy; ^8^ Department of Endocrinology and Clinical Nutrition University Hospital Zurich and University of Zurich Zurich Switzerland; ^9^ Inherited Kidney Diseases, Nephrology Department, Fundació Puigvert, Institut d'Investigacions Biomèdiques (IIB‐Snt Pau), Department of Medicine Universitat Autònoma de Barcelona Barcelona Spain; ^10^ M.A.G.I.C. (Metabolics and Genetics in Canada) Clinic Ltd. Calgary Alberta Canada; ^11^ Cardiology Department, Reference Center on Lysosomal Storage Disorders, Hospital Senhora da Oliveira Guimarães Portugal; ^12^ Department of Medical Genetics University of British Columbia Vancouver British Columbia Canada; ^13^ Second Department of Internal Cardiovascular Medicine, First Faculty of Medicine Charles University and General University Hospital Prague Czech Republic; ^14^ Amicus Therapeutics, Inc. Princeton New Jersey USA; ^15^ University Hospital Würzburg Würzburg Germany

**Keywords:** Fabry disease, migalastat, real world evidence

## Abstract

Fabry disease is a progressive, X‐linked lysosomal disorder caused by reduced or absent α‐galactosidase A activity due to *GLA* variants. The effects of migalastat were examined in a cohort of 125 Fabry patients with migalastat‐amenable *GLA* variants in the followME Pathfinders registry (EUPAS20599), an ongoing, prospective, patient‐focused registry evaluating outcomes for current Fabry disease treatments. We report annualised estimated glomerular filtration rate (eGFR) and Fabry‐associated clinical events (FACEs) in a cohort of patients who had received ≥3 years of migalastat treatment in a real‐world setting. As of August 2022, 125 patients (60% male) had a mean migalastat exposure of 3.9 years. At enrolment, median age was 58 years (males, 57; females, 60) with a mean eGFR of 83.7 mL/min/1.73 m^2^ (*n* = 122; males, 83.7; females, 83.8) and a median left ventricular mass index of 115.1 g/m^2^ (*n* = 61; males, 131.2; females, 98.0). Mean (95% confidence interval) eGFR annualised rate of change in the overall cohort (*n* = 116) was −0.9 (−10.8, 9.9) mL/min/1.73 m^2^/year with a similar rate of change observed across patients with varying levels of kidney function at enrolment. Despite population age and baseline morbidity, 80% of patients did not experience a FACE during the mean 3.9 years of migalastat exposure. The incidence of renal, cardiac, and cerebrovascular events was 2.0, 83.2, and 4.1 events per 1000 patient‐years, respectively. These data support a role of migalastat in preserving renal function and multisystem effectiveness during ≥3 years of migalastat treatment in this real‐world Fabry population.

## 1 INTRODUCTION

Fabry disease is a rare, multisystem, progressive X‐linked lysosomal disorder caused by the functional deficiency of α‐galactosidase A enzyme due to *GLA* gene variants.^1^, ^2^ Enzyme deficiency gives rise to lysosomal dysfunction which occurs within many cells and tissues throughout the body with many patients demonstrating abnormal levels of glycosphingolipids, predominantly globotriaosylceramide and globotriaosylsphingosine.^1^, ^2^ This results in multisystem organ involvement and a wide variety of clinical symptoms and manifestations, including cardiovascular disease (CVD), stroke, end‐stage renal disease, and shorter life expectancy.^3^

The type and the age of onset of Fabry disease manifestations vary, with Fabry disease exhibiting a wide spectrum of clinical presentations with varying degrees of disease severity and organ involvement that affects both sexes.^3^ Patients typically present with early‐ or later‐onset disease.^1^, ^4^ Early‐onset disease, also known as ‘classical phenotype’ and associated with little to no enzyme production in males, presents with symptoms during childhood that progress to multiorgan failure.^1^, ^4^, ^5^ Later‐onset Fabry disease has more variable disease progression in which patients commonly experience a slower decline in renal function compared with classic males.^6^, ^7^ As such, delays in diagnosis are common, with cardiac symptoms, proteinuria, and/or decreased estimated glomerular filtration rate (eGFR) typically presenting in adulthood.^1^, ^8^ By the age of 40 years, patients typically experience more severe disease and higher incidence of clinical events with disease burden continuing to increase with age.^9^ CVD is the current leading cause of death in patients with Fabry disease across phenotypes, and patients with Fabry disease have a higher risk for cardiac events relative to the general population.^10^, ^11^, ^12^, ^13^

Currently approved treatments for Fabry disease include intravenously delivered enzyme replacement therapy (ERT) with either agalsidase alfa, beta, or pegunigalsidase alfa, or oral treatment with migalastat, a first‐in‐class pharmacological chaperone treatment for patients with migalastat‐amenable *GLA* variants. Migalastat has been shown to stabilise eGFR and significantly decrease left ventricular mass index (LVMi) in clinical trials, and their open‐label extension studies, as well as in real‐world studies.^14^, ^15^, ^16^, ^17^, ^18^, ^19^, ^20^ However, data on the effectiveness of long‐term migalastat treatment in a real‐world clinical setting are limited in number and by cohort size.^19^

The followME Pathfinders registry (EUPAS20599) is an ongoing patient‐focused registry evaluating real‐world safety, effectiveness, and patient‐reported outcomes for current Fabry disease treatments, with a focus on patients receiving migalastat.^21^ The occurrence of serious adverse events (SAEs) and Fabry‐associated clinical events (FACEs), which are key indicators of safety and effectiveness, and include cardiac, cerebrovascular, and renal events, and overall survival, will be evaluated over a period of 5 years in this study. Although early in the analysis phase of the registry, this cohort of 125 patients in the followME registry represents a population with clinically significant Fabry disease in which to assess real‐world migalastat use and is the most comprehensive data set following the approval of migalastat for the treatment of Fabry disease.^22^

Here, we present renal function data, and incidence and prevalence of FACEs as a measure of multisystem effectiveness in a cohort of 125 patients with migalastat‐amenable *GLA* variants^23^ who received migalastat for at least 3 years after enrolment into the followME Pathfinders registry.

## 2 METHODS

### 2.1 Registry data collection

The followME Fabry Pathfinders registry (EUPAS20599) is an ongoing prospective, international, observational programme of patients with Fabry disease in 59 centres. At the time of data cutoff, 57 sites had at least one patient enrolled. Patients can be treated with migalastat or ERT, or receive no treatment, and all patients are followed up for 5 years after enrolment. Up to 2 years of retrospective data are included in this analysis, depending on treatment start date. All patients provided written informed consent for participation in this study. The study was conducted in accordance with the ethical principles of the Declaration of Helsinki and Good Pharmacoepidemiology Practice guidelines, along with applicable privacy laws and local regulations for each participating site.

### 2.2 Patients and study design

**FIGURE 1:**
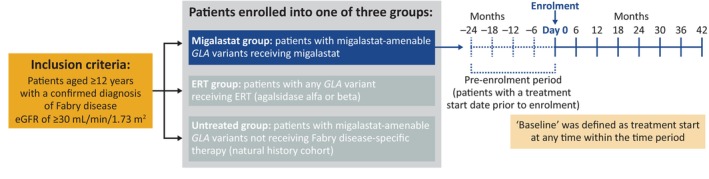
Study design. eGFR, estimated glomerular filtration rate; ERT, enzyme replacement therapy.

Patients ≥12 years old with a confirmed diagnosis of Fabry disease, eGFR ≥30 mL/min/1.73 m^2^ at enrolment and who had not received any treatment or had received up to 2 years of prior treatment were enrolled into one of three groups: migalastat, ERT (agalsidase alfa or beta), or untreated. Patients were included in the current analysis if they had migalastat‐amenable *GLA* variants, had initiated migalastat treatment no more than 24 months prior to enrolment, and had at least 3 years of migalastat exposure as of August 2022 (Figure 1). Full inclusion criteria can be found in Appendix S1. Due to the need for baseline matching adjustments to enable comparisons across groups, data for the untreated and ERT‐treated groups were not included at this time.

### 2.3 Registry procedures

This registry is a non‐interventional study; patients are observed under local treatment practice and no study‐specific visits, tests, or clinical assessments are required. For all patients, clinical treatment decisions were made independently from the decision to enrol the patient into the registry. Treatment switching was permitted in the study and determined based on ongoing medical care and was not influenced by participation in the registry. Only patients with amenable variants could be switched to migalastat treatment. Clinical events were assessed as FACEs by the investigator based on local treatment practice. If a patient discontinued the registry due to an SAE or had an ongoing SAE at the end of the registry follow‐up period, the patient was followed up until the SAE resolved. Patients who exited the study were observed for safety for 30 days after their last migalastat dose.

### 2.4 Outcomes and assessments

Migalastat multisystem effectiveness was evaluated through the incidence and prevalence of FACEs which included renal events, cardiovascular events, cerebrovascular events, and/or death due to FACEs.^9^ Full definitions of FACEs can be seen in Table 1.^24^ Other effectiveness variables in this analysis included eGFR Chronic Kidney Disease Epidemiology Collaboration (eGFR_CKD‐EPI_) annualised change based on serum creatinine measurements. Due to the limited collection of LVMi data up to the time of the data cut, LVMi data has not yet been analysed and was not included. Neither eGFR_CKD‐EPI_ slope per se or LVMi change were included in the definition of a renal or cardiac FACE. Multiorgan involvement was defined as the involvement of ≥2 of the following organ systems: renal, cardiac, central nervous, peripheral nervous, and gastrointestinal. Involvement of an organ system was based on a medical chart review of the following signs/symptoms of Fabry disease: acroparesthesia, gastrointestinal symptoms, hearing loss, and magnetic resonance imaging brain changes using medical history, as well as eGFR <90 mL/min/1.73 m^2^ and LVMi ≥95 g/m^2^ (females) or ≥115 g/m^2^ (males) at enrolment.

**TABLE 1:** Definition of Fabry‐associated clinical events (FACEs).

	Definitions
**Cardiac clinical events**	Myocardial infarction New symptomatic arrhythmia requiring medication, direct current cardioversion, or interventional procedure (e.g., ablation, pacemaker, or defibrillator implantation) Unstable angina defined by national practice guidelines and accompanied by electrocardiographic changes resulting in hospitalisation^a^ Congestive heart failure requiring hospitalisation Any major cardiac medical procedure (e.g., valve replacement, stent implantation, and transplant)
**Cerebrovascular clinical events**	Stroke (documented by a physician) Transient ischaemic attack (documented by a physician)
**Renal clinical events**	Doubling of serum creatinine level from the start of baseline (where levels remained double or greater between two consecutive visits)^48^ End‐stage renal disease requiring long‐term dialysis or transplantation
**Death**	Death due to FACEs^b^

^a^ As reported by investigators based on local treatment practice.

^b^ Analysed separately but reported within the relevant category of cardiac, cerebrovascular, or renal clinical events.

### 2.5 Statistical methods

Due to the study design, no formal hypothesis testing was performed. Demographic data, disease characteristics at enrolment and at start of treatment, and medical history were summarised. FACEs are presented both as exposure‐adjusted incidence rates (calculated per 1000 patient‐years) and prevalence (percentage of patients experiencing an event). Annualised rate of change in eGFR_CKD‐EPI_ for the three eGFR_CKD‐EPI_ categories at enrolment were compared using one‐way analysis of variance.

For longitudinal assessments, data are anchored at enrolment (day 0). Analysis was performed to account for patients starting migalastat at different time points prior to their enrolment into the registry and all available retrospective data are included in this analysis. The baseline was defined as treatment initiation at any time within the study period (i.e., starting from 24 months prior to enrolment onward). For patients included in the analysis with a treatment start date prior to enrolment, the treatment start date was considered baseline (Figure 1) and all on‐migalastat data are included in this analysis. Retrospective eGFR data were available for 62 patients who had a treatment start date prior to enrolment. For patients included in the analysis who did not have a treatment start date prior to enrolment, all retrospective data from the prior 24 months were included for descriptive purposes, but no pre‐enrolment baseline was defined.

Data were not adjusted for any differential selection bias. Prior clinical events were defined as those captured in the medical history and that had occurred before migalastat treatment initiation. To prevent undercounting, events without a date were attributed to the migalastat treatment period. Various subgroups of interest were analysed to provide context for the data and allow for comparison with other analyses in the literature.

## 3 RESULTS

### 3.1 Patient demographics and disease characteristics at enrolment

As of August 2022, 125 patients enrolled into the followME Pathfinders registry who had migalastat‐amenable *GLA* variants and at least 3 years of migalastat exposure were included in this analysis. Patients were enrolled from Austria (*n* = 6), Czech Republic (*n* = 8), Canada (*n* = 15), Germany (*n* = 26), Hungary (*n* = 1), Italy (*n* = 16), Portugal (*n* = 8), Switzerland (*n* = 6), Spain (*n* = 14), and the United Kingdom (*n* = 25). Of these, 60% (*n* = 75) were male, and the overall median age of patients was 58.0 years, with 81.6% of patients over 40 years of age (Table 2). Of patients who switched treatment, prior to enrolment, 29 patients switched to migalastat from an ERT and post‐enrolment, three patients switched to migalastat from ERT, and one patient switched to ERT from migalastat. Patients had a mean migalastat exposure of 3.9 years and a total exposure to migalastat of 493 patient‐years. Prior to enrolment, 116 patients had received a mean of 1.0 years of migalastat exposure, and nine patients initiated migalastat at or after enrolment.

**TABLE 2:** Patient demographics and disease characteristics at enrolment.

	Overall	Males	Females
*n* (%)	125 (100)	75 (60.0)	50 (40.0)
**Age (years)**			
Median (range)	58.0 (16.0–77.0)	57.0 (16.0–75.0)	60.0 (18.0–77.0)
>40 years, *n* (%)	102 (81.6)	59 (78.7)	43 (86.0)
≤40 years, *n* (%)	23 (18.4)	16 (21.3)	7 (14.0)
**Race, *n* (%)**			
Caucasian	124 (99.2)	74 (98.7)	50 (100)
Asian	1 (0.8)	1 (1.3)	0 (0.0)
**Ethnicity, *n* (%)**			
Hispanic or Latino	23 (18.4)	12 (16.0)	11 (22.0)
Not Hispanic or Latino	80 (64.0)	52 (69.3)	28 (56.0)
Not reported	22 (17.6)	11 (14.7)	11 (22.0)
**Top three amenable variants, *n* (%)**			
p.N215S	38 (30.4)	32 (25.6)	6 (4.8)
p.S238N	10 (8.0)	7 (5.6)	3 (2.4)
p.F113L	9 (7.2)	9 (7.2)	0 (0.0)
**eGFR_CKD‐EPI_, mL/min/1.73 m^2^** ^a^			
*n* (%)	122 (97.6)	72 (96.0)	50 (100)
Mean (SD)	83.7 (22.5)	83.7 (24.2)	83.8 (20.1)
eGFR ≥90	46 (36.8)	29 (38.7)	17 (34.0)
eGFR ≥60 to <90	59 (47.2)	32 (42.7)	27 (54.0)
eGFR ≥30 to <60	16 (12.8)	11 (14.7)	5 (10.0)
eGFR ≥20 to <30	1 (0.8)	0 (0.0)	1 (2.0)
Missing	3 (2.4)	3 (4.0)	0 (0.0)
**uACR, mg/g**			
*n* (%)	40 (32.0)	23 (30.7)	17 (34.0)
Median (range)	19.0 (0–1124)	14.0 (0–312)	26.6 (0–1124)
**LVMi, g/m^2^**			
*n* (%)	61 (48.8)	34 (45.3)	27 (54.0)
Median (range)	115.1 (23.9–289.0)	131.2 (43.0–289.0)	98.0 (23.9–209.0)

Abbreviations: CKD‐EPI, Chronic Kidney Disease Epidemiology Collaboration; eGFR, estimated glomerular filtration rate; LVMi, left ventricular mass index; SD, standard deviation; uACR, urine albumin creatinine ratio.

^a^ One female patient with an eGFR value ≥20 to <30 at enrolment met the inclusion criteria and was included in the study; however, treatment of this patient is considered off‐label. Three patients were excluded from the analysis population as they were ineligible to participate in the registry. These patients had physiologically implausible recorded eGFR values due to incorrect entry of data or an eGFR at enrolment of much less than 30 mL/min/1.73 m^2^. Data will continue to be collected for these patients for future analyses.

**FIGURE 2:**
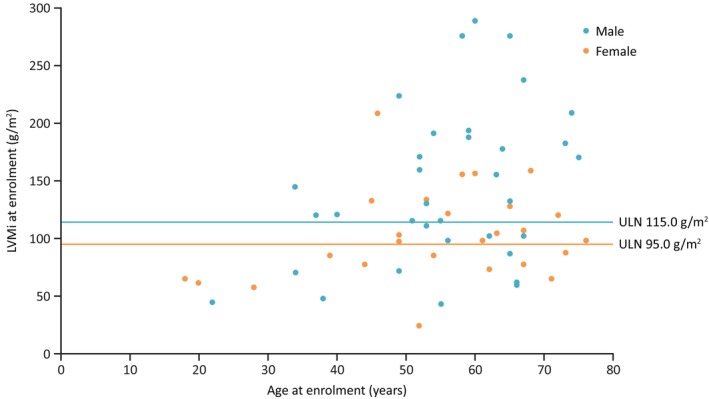
LVMi by age at enrolment separated by males and females. The reference values for LVMi were based on the M mode linear method to avoid over attribution to LVH. Reference values for the 2D method are: females, 88 g/m^2^ and males, 102 g/m^2^. LVH, left ventricular hypertrophy; LVMi, left ventricular mass index; ULN, upper limit of normal.

Disease characteristics at enrolment are detailed in Table 2. Mean eGFR_CKD‐EPI_ for the overall population (*n* = 122) was 83.7 mL/min/1.73 m^2^, with 12.8% (*n* = 16) of patients experiencing renal impairment with an eGFR of <60 mL/min/1.73 m^2^. Although urine albumin creatinine ratio (uACR) was only reported for a small number of patients, females had a higher median uACR than males at enrolment (26.6 mg/g [*n* = 17] vs.14.0 mg/g [*n* = 23], respectively). Of patients with available LVMi data at enrolment, median LVMi for the overall population was 115.1 g/m^2^. LVMi was above the upper limit of normal (ULN) indicating left ventricular hypertrophy (LVH) in both sexes (median LVMi in males [*n* = 34]: 131.2 g/m^2^ [ULN: 115.0 g/m^2^]; females [*n* = 27]: 98.0 g/m^2^ [ULN: 95.0 g/m^2^]),^25^ representing 64.7% of males and 59.3% of females (Figure 2). In patients >40 years at enrolment, mean eGFR_CKD‐EPI_ was lower (77.5 mL/min/1.73 m^2^, *n* = 100) and median LVMi was higher (120.1 g/m^2^, *n* = 51) than the overall population and compared with patients ≤40 years (mean eGFR_CKD‐EPI_: 112.1 mL/min/1.73 m^2^, *n* = 22; median LVMi: 67.5 g/m^2^, *n* = 10).

**FIGURE 3:**
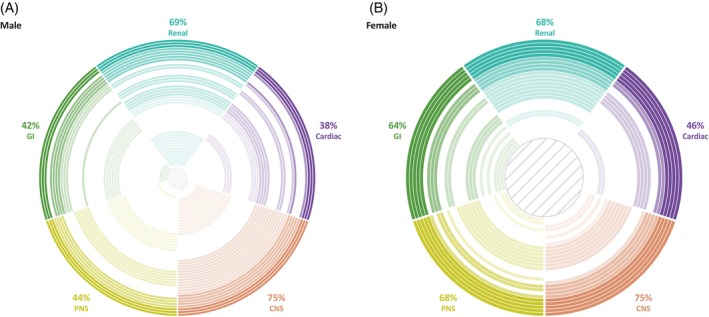
Multiorgan involvement by sex. Each ring represents one patient with involvement of ≥2 organ systems. Each colour represents the involvement of an organ: the darker the shade, the more organ systems were involved (up to five) for that patient. (A) There are 48 rings, each representing one male patient with involvement of ≥2 organ systems. (B) There are 28 rings, each representing one female patient with involvement of ≥2 organ systems. The proportion of patients with involvement of each organ system is shown only for patients with involvement of ≥2 organ systems and is not indicative of the proportion of patients with organ involvement for the overall population. CNS, central nervous system; GI, gastrointestinal; PNS, peripheral nervous system.

Overall, multiorgan involvement (involvement of ≥2 organ systems) was reported in 60.8% of patients (56.0% [28/50] of females and 64.0% [48/75] of males; Figure 3).

### 3.2 Renal measures

**FIGURE 4:**
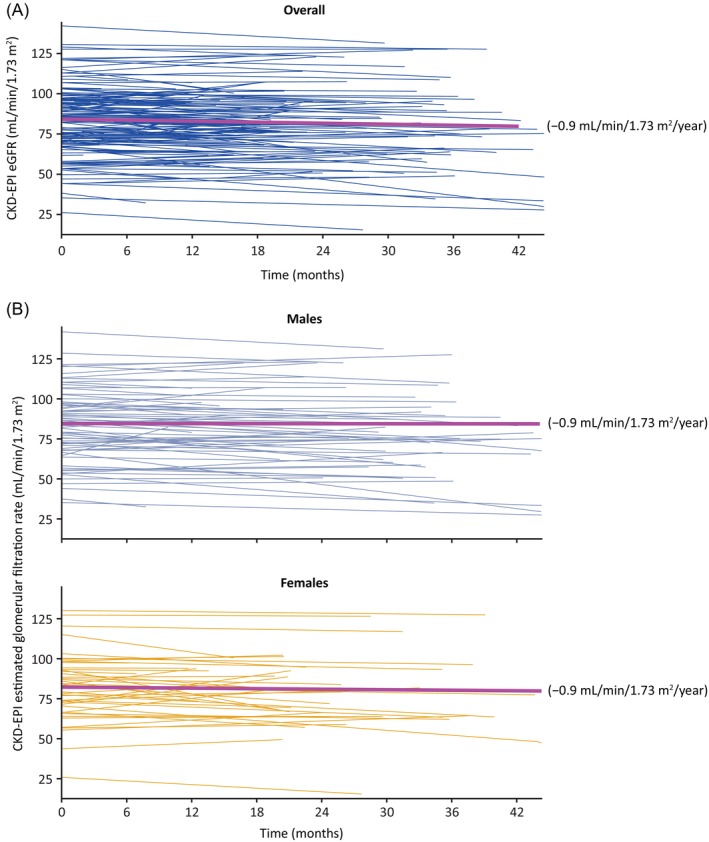
Annualised rate of change in eGFR_CKD‐EPI_ by patient for (A) the overall patient population and (B) by sex. Solid magenta line indicates the mean eGFR_CKD‐EPI_ annualised rate of change. Annualised rate of eGFR change was calculated using three or more data points for 91/116 patients and two data points for 25/116 patients. The mean (95% CI) for (A) the overall patient population was −0.9 (−10.8, 9.9) mL/min/1.73 m^2^/year and (B) for males were −0.9 (−9.8, 13.3) mL/min/1.73 m^2^/year and for females were −0.9 (−11.0, 9.9) mL/min/1.73 m^2^/year. One female patient with an eGFR value ≥20 to <30 at enrolment met the inclusion criteria and was included in the study; however, treatment of this patient is considered off‐label. CI, confidence interval; CKD‐EPI, Chronic Kidney Disease Epidemiology Collaboration; eGFR, estimated glomerular filtration rate.

The mean (95% confidence interval [CI]) annualised rate of change in eGFR_CKD‐EPI_ for the overall population (*n* = 116) was −0.9 (−10.8, 9.9) mL/min/1.73 m^2^/year (Figure 4A) during the mean 3.9 years of migalastat exposure. A similar mean (95% CI) annualised rate of change was observed for both males (−0.9 [−9.8, 13.3] mL/min/1.73 m^2^/year, *n* = 70) and females (−0.9 [−11.0, 9.9] mL/min/1.73 m^2^/year, *n* = 46; Figure 4B).

**FIGURE 5:**
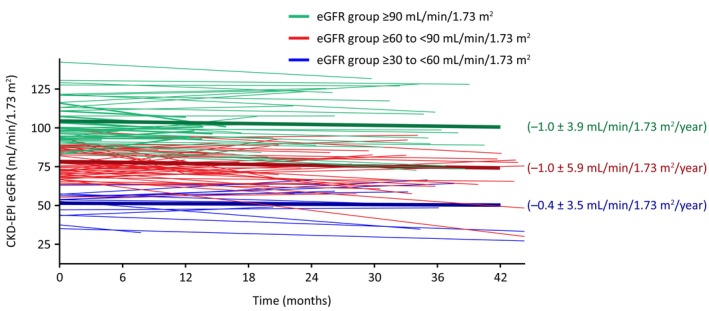
Annualised rate of change in eGFR_CKD‐EPI_ for each patient by eGFR category at enrolment. Dark solid lines indicate the mean eGFR_CKD‐EPI_ annualised rate of change. Annualised rate of eGFR_CKD‐EPI_ change was calculated using three or more data points for 91/116 patients and two data points for 25/116 patients. The 95% CI for eGFR group ≥90 mL/min/1.73 m^2^ at enrolment was −5.9, 8.3 mL/min/1.73 m^2^/year, for eGFR group ≥60 to <90 mL/min/1.73 m^2^ at enrolment was −10.8, 11.3 mL/min/1.73 m^2^/year, and for eGFR group ≥30 to <60 mL/min/1.73 m^2^ at enrolment was −7.5, 4.6 mL/min/1.73 m^2^/year. CI, confidence interval; CKD‐EPI, Chronic Kidney Disease Epidemiology Collaboration; eGFR, estimated glomerular filtration rate.

When examining across eGFR categories at enrolment, a statistically comparable, stable mean annualised rate of change in eGFR_CKD‐EPI_ was seen (*p* = 0.692; Figure 5). Mean ± standard deviation (SD) annualised eGFR_CKD‐EPI_ change in patients with an eGFR ≥90 mL/min/1.73 m^2^ was −1.0 ± 3.9 mL/min/1.73 m^2^/year, −1.0 ± 5.9 mL/min/1.73 m^2^/year in patients with an eGFR ≥60 to <90 mL/min/1.73 m^2^ and − 0.4 ± 3.5 mL/min/1.73 m^2^/year in patients with an eGFR ≥30 to <60 mL/min/1.73 m^2^.

### 3.3 Incidence of FACEs

During the mean 3.9 years of migalastat exposure, 100 patients (80.0%) did not experience a FACE. The most common FACE was a cardiac event, which was experienced in 24 patients (19.2%). One patient (0.8%) experienced a renal event (doubling of serum creatinine level); eGFR_CKD‐EPI_ of this patient was 60.4 mL/min/1.73 m^2^ at enrolment. Two patients (1.6%) experienced a cerebrovascular event.

Prior to initiating migalastat, nine patients (7.2%; five female, four male) each experienced one FACE (6 cerebrovascular events, 3 cardiac events, and 0 renal events). During the migalastat treatment period, 14.4% (*n* = 18) of patients experienced one FACE and 5.6% (*n* = 7) of patients (three female, four male) experienced multiple events (range: 2–8 events), 57.1% (*n* = 4) of whom had experienced a FACE prior to migalastat initiation. One patient experienced eight symptomatic arrhythmia events while on migalastat treatment that required either medication, direct current cardioversion, or interventional procedure. In total, 44 events were reported during migalastat treatment and were included in this analysis. All events are shown by patient in Table S1.

**FIGURE 6:**
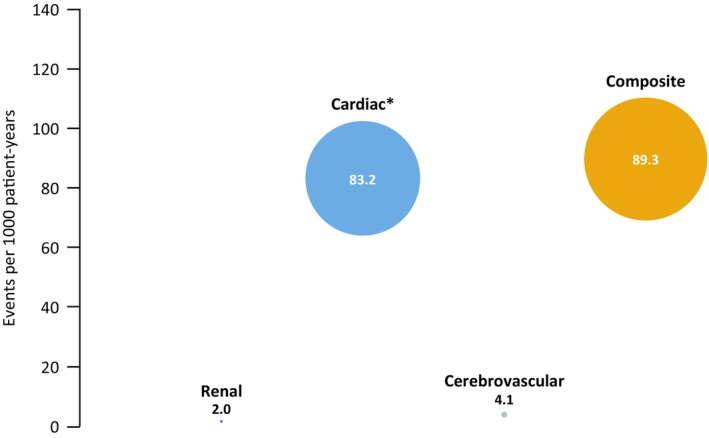
Incidence of FACEs per 1000 patient‐years in patients who received migalastat. *Includes one death due to a cardiac event. FACE, Fabry‐associated clinical event.

Overall, the incidence for composite events (includes all individual cerebrovascular, cardiac, and renal FACEs and death combined) was 89.3 events per 1000 patient‐years (Figure 6). One death due to cardiac failure was reported in a male patient at 30 months post‐enrolment; the patient was 66 years old at enrolment and had the p.N215S variant. [Correction added on 13 August 2024, after first online publication: The variant in the preceding sentence has been corrected in this version.] He was diagnosed with Parkinson disease in 2009 and Fabry disease in 2018. He had no previous history of FACEs before enrolment.

### 3.4 Incidence of serious adverse events

Overall, 16.0% (*n* = 20) of patients experienced 29 SAEs while on migalastat; five patients had multiple SAEs. One SAE owing to a transient ischaemic attack was deemed by the treating physician as possibly related to migalastat, three SAEs (atrial fibrillation, myocardial infarction, and cardiac failure) were deemed unlikely to be related, and the rest were determined to be unrelated to migalastat. A list of all SAEs can be found in Table S2. Of the 29 SAEs, 14 were considered FACEs and have been included in the FACE prevalence/incidence.

### 3.5 Fabry disease and migalastat effectiveness in patients without the p.N215S variant

The three most common amenable variants observed in this patient population were p.N215S (30.4%), p.S238N (8.0%), and p.F113L (7.2%) and were all most frequently represented in males compared with females (Tables 2; S3). A greater variety of amenable variants was observed in the female population compared to males (30 variants in 49 females vs. 20 variants in 75 males) in this group. The most common amenable variants by sex were p.N215S (25.6%), p.F113L (7.2%), and p.S238N (5.6%) in males and p.N215S (4.8%), p.R356W (3.2%) and p.A143T, p.P205T, p.R301Q, and p.S238N (all 2.4%) in females (Table S3). A total of seven patients (5.6%) had variants of uncertain significance (VUSs); four patients had the p.A143T variant and three patients had the p.R118C variant.

Due to the high proportion of patients with the p.N215S variant in this cohort, and the known cardiac pathology of p.N215S, a subanalysis was performed to assess the effectiveness of migalastat in patients without the p.N215S variant. Overall, 87 patients (69.6%) had a variant other than the p.N215S variant, of whom 50.6% were female. At enrolment, the non‐p.N215S group was broadly comparable to the overall study cohort (patient demographics and disease characteristics can be seen in Tables S4 and 2, respectively), with similar age (median 56 vs. 58 years, respectively), mean ± SD eGFR_CKD‐EPI_ (85.5 ± 24.1 [*n* = 86] vs. 83.7 ± 22.5 mL/min/1.73 m^2^ [*n* = 122]), median LVMi (115.3 [*n* = 48] vs. 115.1 g/m^2^ [*n* = 61]) and proportion of patients with multiorgan involvement (62.1% vs. 60.8%). In comparison to the non‐p.N215S group, the p.N215S group at enrolment (*n* = 38) was older (median 60.5 years) with a higher proportion of male patients (84.2%) and a longer duration of migalastat treatment prior to enrolment (median 17.3 vs. 9.8 months). Patients in the p.N215S group also had a lower median LVMi (102.0 vs. 115.3 g/m^2^, respectively) and a higher proportion of patients had an eGFR 60 to <90 mL/min/1.73 m^2^ (57.9% vs. 42.5%) at enrolment compared to the non‐p.N215S group.

The median annualised rate of change in eGFR_CKD‐EPI_ (mL/min/1.73 m^2^/year) in the non‐p.N215S group and the p.N215S group were comparable with the overall population during the 3.9 years of migalastat exposure (−1.5 [Q1 −3.7, Q3 0.8], *n* = 81 vs. −0.7 [Q1 −3.1, Q3 2.8], *n* = 35). Overall, a slightly higher proportion of patients with the p.N215S variant experienced a FACE compared with those without the variant (26.3% [*n* = 10] vs. 17.2% [*n* = 15], respectively). Of patients experiencing a FACE, all events in the p.N215S subgroup were cardiac‐related, while in the non‐p.N215S group, 16.1% (*n* = 14) experienced a cardiac event, 1.1% (*n* = 1) experienced a renal event and 2.3% (*n* = 2) experienced a cerebrovascular event. Patients in the non‐p.N215S and the p.N215S group had a similar incidence of cardiac events compared to the overall population (83.4 and 82.9 events per 1000 patient‐years, respectively vs. 83.2 events) and the non‐p.N215S group also had a similar overall incidence of composite events compared to the overall population (92.4 vs. 89.3 events per 1000 patient‐years, respectively), with different distribution of types of events observed in the two populations, notably renal and cerebrovascular events in the non‐p.N215S group compared to mainly cardiac events in the overall population (Figure 6 and Table S5).

## 4 DISCUSSION

This study used data from the followME Fabry Pathfinders registry to assess the effectiveness of migalastat in patients with Fabry disease and amenable variants who were treated for at least 3 years in a real‐world clinical setting. Long‐term clinical trials of migalastat have shown treatment benefits including stabilised renal function, sustained cardiac efficacy, and low incidence of FACEs up to 8.6 years. The clinical practice data described here aligns with observations from clinical trials and contributes multicentre, multinational clinical findings to the limited data available describing the real‐world effectiveness of migalastat.

This real‐world study consisted of an older patient population compared with previous clinical trials and real‐world evidence studies of migalastat or ERT.^20^, ^25^, ^26^ Enrolment characteristics and medical histories indicated that a similar proportion of males and females had advanced cardiac disease, as indicated by the presence of LVH (Figure 2), and multiorgan involvement was observed in over half of the patients. Overall, 13% of patients had an eGFR <60 mL/min/1.73 m^2^ at enrolment, and of patients with LVMi data at enrolment, 62% had LVMi above the cutoff for LVH.

In relation to the established treatment goal of an annualised decline in eGFR up to −3 mL/min/1.73 m^2^/year in patients with Fabry disease with progressing kidney disease,^26^ the mean annualised change in eGFR in this real‐world cohort was stable (−0.9 [−10.8, 9.9] mL/min/1.73 m^2^/year) over nearly 4 years of migalastat treatment including in patients with the lowest baseline eGFR category. This preservation of renal function aligns with the low renal event rate that was observed in this study and supports the finding of an association of eGFR slope with clinical outcomes over time.^27^ Additionally, this real‐world eGFR data corroborates long‐term clinical trial findings where patients treated with migalastat demonstrated stable eGFR over time, regardless of ERT treatment history.^17^ The rate of change in eGFR in this study is also comparable to the rate of change observed in patients treated with ERT in a real‐world setting^28^, ^29^ and is also consistent with age‐related decline in the general adult population.^30^ It is important to note that eGFR was stable across all eGFR categories, especially considering that 13% of patients had renal impairment at enrolment (eGFR ≥30 to <60 mL/min/1.73 m^2^), suggesting preservation of renal function while on migalastat treatment, regardless of renal involvement at enrolment. Prior real‐world studies of migalastat treatment with fewer patients and shorter duration have reported a decline in eGFR between −1 and −9 mL/min/1.73 m^2^/year after 1–2 years of treatment,^18^, ^19^, ^20^, ^31^, ^32^ though a Delphi consensus as well as health authority guidance recommends at least 2 years of follow‐up for reliable eGFR data.^33^, ^34^ In comparison, the followME Fabry Pathfinders registry is a larger, more geographically diverse population with significant disease burden at enrolment and longer follow‐up, so these findings in the current analysis may be more representative of the treatment effect of migalastat on Fabry disease in the real‐world setting. Future studies comparing eGFR rate of change before and after treatment with migalastat should be carried out to confirm these results, which suggest that migalastat has a role in preserving renal function.

In this real‐world study, 80% of patients did not experience a FACE within nearly 4 years of migalastat treatment. It is also important to highlight that no stroke events were reported in patients during this study despite being 12 times more common in patients with Fabry disease than in the general population.^35^ This is also in contrast to real‐world studies of ERT‐treated patients in which no reduction in the rate of stroke was observed.^36^, ^37^ Additionally, 82% of patients in this study were aged >40 years, a patient population that was generally older than reported in clinical trials of ERT and migalastat and that is associated with a higher FACE incidence.^38^ Comparing the overall FACEs incidence rate of 89.3 events per 1000 patient‐years in this study to the ATTRACT clinical trial data, reanalysed using the current definition of FACEs for both migalastat‐ and ERT‐treated cohorts, patients treated with migalastat for up to 18 months in ATTRACT had a slightly lower incidence rate of 61 events per 1000 patient‐years compared to the incidence rate observed in this real‐world study, while patients continuing ERT up to 18 months of treatment had an incidence rate of 327 events per 1000 patient‐years.^9^ It should be noted that in this real‐world study, all events were counted regardless of whether a patient experienced one or multiple events during the follow‐up period, whereas other Fabry disease studies only count the first event.^38^, ^39^, ^40^ Additionally, in this study, attribution of a clinical event to Fabry disease was made according to the study definitions with discretion of the investigator to state if another cause was evident. However, because there is no standardised definition for FACEs, different definitions and analysis methods have been used in studies of Fabry disease, limiting direct comparisons between studies.^9^ Consensus between experts on a standardised definition of FACEs would make some comparison between studies more manageable and would increase the impact on disease management through better evaluation of treatment outcomes.^9^

Incidence of cardiac events in this real‐world study is numerically higher than observed in clinical trials of patients with Fabry disease.^9^ This could be explained by the older age of our study population where events could have been overattributed to Fabry disease, along with high LVMi and more advanced disease at enrolment. These data are suggestive of a population with a heightened risk of cardiac complications and also aligns with the natural history of Fabry disease, where FACE incidence increases with organ damage and age.^38^ Patients with Fabry disease have been shown to be at higher risk than others with hypertrophic cardiomyopathy for specific cardiac events such as myocardial infarction with non‐obstructive coronary arteries.^12^ Additionally, cardiovascular risk factors increase over time in older populations, as well as atherosclerotic coronary artery disease and degenerative valve disease, and this was not controlled for in the analysis. Furthermore, 30% of patients in this cohort had the p.N215S variant, which has been associated with cardiac pathology and late‐onset cardiac manifestations.^41^ When removing this group from the analysis however, the subanalysis found that the non‐p.N215S and the p.N215S groups had a similar incidence of cardiac events compared to the overall population (Table S5 and Figure 6).

Clinical events in patients with Fabry disease may be caused by or have a contribution from other aetiologies, such as cardiovascular risk factors, coronary/cerebrovascular atherosclerotic artery disease, and degenerative valve disease, which are common among the general population with ageing. Therefore, for any given patient, it may be clinically impossible to be certain that the event is related to Fabry disease. However, in this study, any clinical event that met the prespecified, per‐protocol definition of a cardiac, renal, or cerebrovascular FACE was attributed to migalastat treatment to avoid undercounting of events. It is notable that LVMi assessment was not available in all patients, but those with available values showed a high LVH rate in this study. This may be an ascertainment bias, but it does highlight the need for improved monitoring of cardiac status to ensure timely treatment initiation that may slow disease progression and improve cardiac outcomes.^9^, ^42^, ^43^, ^44^

The older age of patients, mostly male sex, and high proportion with advanced cardiac involvement may be representative of how migalastat is being used in clinical practice. Since *GLA* is on the X chromosome, Mendelian inheritance dictates that two‐thirds of all individuals carrying a *GLA* variant should be female.^45^ This population was 40% female, and these females had significant disease burden at enrolment regardless of prior treatment status.^46^ Females are traditionally underrepresented in ERT clinical trials;^9^, ^47^ therefore, assessing the effectiveness of migalastat in female patients will help support the treatment of females with Fabry disease.

Limitations of this study include the observational nature of data from the registry and the absence of a central source data review for analysis. This analysis is also limited by the incomplete retrospective data and subsequent lack of longitudinal LVMi data which may have been impacted by lower attendance at follow‐up visits due to the COVID pandemic. There may also be potential ascertainment bias from incomplete data or in reporting parameters such as uACR and LVMi among patients with severe disease. Due to the study design and lack of historical data, eGFR rate of change before migalastat treatment could not be analysed in this study and, therefore, a comparison of eGFR slopes before and after migalastat treatment could not be performed to confirm the protective effect of migalastat on renal function. The avoidance of undercounting of clinical events may have also resulted in an over‐attribution of FACEs to migalastat treatment which should be addressed in future studies. Additionally, the influence of comorbidities and medications, such as angiotensin‐converting enzyme inhibitors and angiotensin II receptor blockers, on the outcomes of FACEs were not examined in this older patient population. This cohort of patients included a high proportion of Caucasian patients, which may skew the presentation of Fabry disease. The limited diversity is likely representative of the patients being managed at expert centres, and representation may grow as diagnosis and management of Fabry disease becomes more easily accessible. A total of seven patients (5.6%) in this cohort had VUSs. As VUSs require clinical evaluation of the phenotype to carefully determine whether the variant is causing a clinical effect in the patient, the decision to treat patients with a VUS was based on local treatment practices and clinical evaluation and was made independently of the study. All seven patients were determined to be eligible for treatment by their treating physician; therefore, they should not have influenced the results of this study. Finally, a comparison of early‐ vs. late‐onset variants would be of clinical interest but data collection in this registry‐based population did not allow the generation of robust phenotype information.

## 5 CONCLUSIONS

These data from the followME Pathfinders registry reinforce the multisystem treatment benefits of migalastat, including preserved renal function and low incidence of FACEs in an older, more geographically diverse Fabry population with significant disease. The data presented here, which are representative of the treatment effect of migalastat on Fabry disease in the real‐world setting, align with previous observations from clinical trials and extends the available data supporting the real‐world multisystem effectiveness of migalastat.

## AUTHOR CONTRIBUTIONS

**Derralynn A. Hughes** has contributed to the conception and design, interpretation of data, and revising the article for important intellectual content and is the guarantor for the article. **Gere Sunder‐Plassmann**, **Joseph D. Giuliano**, and **Peter Nordbeck** have contributed to the conception and design, interpretation of data, and revising the article for important intellectual content. **Ana Jovanovic**, **Eva Brand**, **Michael L. West**, **Daniel G. Bichet**, **Antonio Pisani**, **Albina Nowak**, **Roser Torra**, **Aneal Khan**, **Olga Azevedo**, **Anna Lehman**, and **Aleš Linhart** have contributed to the interpretation of data and revising the article for important intellectual content. **Jasmine Rutecki** has contributed to the conception and design and revising the article for important intellectual content. **Eva Krusinska** has contributed to the analysis and interpretation of data and revising the article for important intellectual content.

## FUNDING INFORMATION

This study was funded by Amicus Therapeutics, Inc. Medical writing assistance was provided by Amy Graham, PhD, at Cogent (an AMICULUM® agency), funded by Amicus Therapeutics, Inc. The authors confirm independence from the sponsors; the content of the article has not been influenced by the sponsors.

## CONFLICT OF INTEREST STATEMENT

Derralynn A. Hughes reports grants from Amicus Therapeutics, Inc., consulting fees from Amicus Therapeutics, Inc., Freeline, Idorsia, Protalix/Chiesi, Sanofi, Takeda and Sangamo Therapeutics, Inc., honoraria from Amicus Therapeutics, Inc., Freeline, Idorsia, Protalix/Chiesi, Sanofi and Takeda, and other support from Chiesi, Freeline, Sanofi, Takeda and Amicus Therapeutics, Inc. Gere Sunder‐Plassmann reports advisory board participation for Amicus Therapeutics, Inc., Chiesi and Sanofi, research grants/funding from Amicus Therapeutics, Freeline, Idorsia and Takeda, honoraria from Amicus Therapeutics, Inc., Chiesi, and Sanofi, and other financial support from Amicus Therapeutics, Inc., Chiesi and Sanofi. Ana Jovanovic reports advisory board participation from Amicus Therapeutics, Inc. and Sanofi, research grants and honoraria from Amicus Therapeutics, Inc. and Takeda, and other financial support from Amicus Therapeutics, Inc. Eva Brand reports research grants, consulting fees, advisory board and speaker honoraria from Amicus Therapeutics, Inc., Chiesi, Sanofi Genzyme and Takeda. Michael L. West reports research grants from Takeda, Sanofi, Alexion, Chiesi and AvroBio, consulting fees from Takeda, advisory board participation for Amicus Therapeutics, Inc. and Sanofi, leadership or fiduciary board/committee roles for Sanofi and Takeda, honoraria from Takeda, Sanofi, Sumitomo and Amicus Therapeutics, Inc., payment for expert testimony from Takeda and Amicus Therapeutics, Inc., and other financial support from Amicus Therapeutics, Inc. Daniel G. Bichet reports advisory board participation, honoraria and other financial support from Amicus Therapeutics, Inc. and Sanofi/Genzyme. Antonio Pisani declares no conflicts of interest. Albina Nowak reports advisory board, speaker honoraria and other financial support from Amicus Therapeutics, Inc., Sanofi Genzyme and Takeda. Roser Torra reports advisory board participation, consulting fees and honoraria for Amicus Therapeutics, Inc., Chiesi, Sanofi and Takeda. Aneal Khan reports research support from Amicus Therapeutics, Inc. Olga Azevedo reports financial support from Takeda, Amicus Therapeutics, Inc., Chiesi and Sanofi Genzyme. Anna Lehman reports research grants from Idorsia, Sanofi Genzyme and Amicus Therapeutics, Inc., consulting fees and advisory board honoraria from Takeda, Sanofi Genzyme and Amicus Therapeutics, Inc., and honoraria from Sanofi Genzyme. Aleš Linhart reports consulting fees, honoraria, advisory board membership and other financial support from Amicus Therapeutics, Inc., Chiesi, Sanofi and Takeda. Jasmine Rutecki and Joseph D. Giuliano are employees and shareholders of Amicus Therapeutics, Inc. Eva Krusinska reports working as a consultant under the contract of Pharmaland Consulting Group. Peter Nordbeck reports consulting and speaker fees from Abiomed, Amicus Therapeutics, Inc., Bayer, Boehringer Ingelheim, Boston Scientific, Cardiac Dimensions, Chiesi, Daiichi Sankyo, Idorsia, Sanofi/Genzyme and Shire/Takeda.

## INFORMED CONSENT

All procedures followed were in accordance with the ethical standards of the responsible committee on human experimentation (institutional and national) and with the Helsinki Declaration of 1975, as revised in 2000 (5). Informed consent was obtained from all patients for being included in the study.

## ETHICAL APPROVAL

The study was conducted in accordance with the ethical principles of the Declaration of Helsinki and Good Pharmacoepidemiology Practice guidelines, along with applicable privacy laws and local regulations for each participating site.

## Supporting information


**APPENDIX S1:** Supporting information.

## Data Availability

Data are available on reasonable request. Requests for access to data may be submitted to Amicus Therapeutics, Inc.
