# Supplementary material for: Renal and multisystem effectiveness of 3.9 years of migalastat in a global real‐world cohort: Results from the followME Fabry Pathfinders registry
Source: J Inherit Metab Dis. 2024 Jul 19;48(1):e12771. doi: 10.1002/jimd.12771 (PMC11730455; doi:10.1002/jimd.12771)
Supplement: Supplementary file 1 — APPENDIX S1: Supporting information. [file JIMD-48-0-s001.docx]

# SUPPLEMENT

# Renal and multisystemic effectiveness of 3.9 years of migalastat in a global real-world cohort: results from the followME Fabry Pathfinders registry

Derralynn A. Hughes, Gere Sunder-Plassmann, Ana Jovanovic, Eva Brand, Michael L. West, Daniel G. Bichet, Antonio Pisani, Albina Nowak, Roser Torra, Aneal Khan, Olga Azevedo, Anna Lehman, Aleš Linhart, Jasmine Rutecki, Joseph D. Giuliano, Eva Krusinska, Peter Nordbeck

Methods

Patients met the following inclusion criteria to be included in the registry:

1. Fabry disease patients 16 years or older who have commenced commercial migalastat treatment within 24 months preceding enrolment, who have eGFR_CKD-EPI_ greater than 30 mL/min/1.73 m^2^ within 12 months prior to enrolment, and are still taking migalastat at the time of enrolment, or who are starting migalastat at the time of enrolment, excluding those who participated in a prior migalastat clinical trial.

Or

2. Fabry disease patients who have commenced commercial migalastat within 24 months preceding enrolment, who do not meet inclusion criteria 1, and are still taking migalastat at the time of enrolment.

Or

3. Fabry disease patients 16 years or older who have commenced commercial migalastat treatment greater than 24 months preceding enrolment, who have eGFR_CKD-EPI_ greater than 30 mL/min/1.73 m^2^ within 12 months prior to enrolment and are still taking migalastat at the time of enrolment, excluding those who participated in a prior migalastat clinical trial.

Or

4. Fabry disease patients 16 years or older who had commenced treatment with migalastat while in a clinical trial, who have eGFR_CKD-EPI_ greater than 30 mL/min/1.73 m^2^ within 12 months prior to enrolment, and who are still taking migalastat at the time of enrolment, having switched to commercial product.

## **Supplementary Table 1** Patients with FACEs prior to and during migalastat treatment.

| **Patient** | **Events prior to treatment** | **Events during treatment** |
| --- | --- | --- |
| 1 | **Cerebrovascular:** Stroke |  |
| 2 |  | **Renal:** Doubling from baseline of serum creatinine on two consecutive visits |
| 3 |  | **Cardiac:** Symptomatic arrhythmia requiring interventional procedure: Defibrillator Implantation |
| 4 | **Cerebrovascular:** Stroke |  |
| 5 |  | **Cardiac:** Symptomatic arrhythmia requiring interventional procedure: Pacemaker |
| 6 |  | **Cardiac:** Symptomatic arrhythmia requiring interventional procedure: Coronary Angiography  **Cardiac:** Myocardial infarction requiring interventional procedure: Stent Implantation |
| 7 |  | **Cardiac:** Symptomatic arrhythmia requiring medication: Oral anticoagulation |
| 8 |  | **Cardiac:** Congestive heart failure requiring hospitalisation or accompanied by elevated B-type natriuretic peptide |
| 9 | **Cerebrovascular:** TIA | **Cerebrovascular:** TIA  **Cardiac:** Symptomatic arrhythmia requiring direct current cardioversion |
| 10 | **Cerebrovascular:** TIA | **Cardiac:** Symptomatic arrhythmia requiring interventional procedure: Preventive ICD recommended  **Cardiac:** Symptomatic arrhythmia requiring interventional procedure: Preventive ICD recommended |
| 11 | **Cerebrovascular:** TIA |  |
| 12 |  | **Cardiac:** Symptomatic arrhythmia requiring interventional procedure: Pacemaker |
| 13 | **Cerebrovascular:** Stroke | **3 Cardiac:** Symptomatic arrhythmia requiring direct current cardioversion  **Cardiac:** Symptomatic arrhythmia requiring interventional procedure: Ablation  **Cardiac:** Major cardiac medical procedure: Stent Implantation  **Cardiac:** Major cardiac medical procedure: Implantation mitral clip in mitral valve  **Cerebrovascular:** TIA |
| 14 |  | **Cardiac:** Symptomatic arrhythmia requiring interventional procedure: Pacemaker |
| 15 | **Cardiac:** Symptomatic arrhythmia requiring direct current cardioversion | **6 Cardiac:** Symptomatic arrhythmia requiring direct current cardioversion  **Cardiac:** Symptomatic arrhythmia requiring interventional procedure: Ablation  **Cardiac:** Symptomatic arrhythmia requiring medication: Oral beta-blocker |
| 16 |  | **Cardiac:** Symptomatic arrhythmia requiring interventional procedure: Pacemaker |
| 17 |  | **Cardiac:** Symptomatic arrhythmia requiring interventional procedure: Pacemaker |
| 18 |  | **Cardiac:** Major cardiac medical procedure: Primary prevention ICD implanted |
| 19 |  | **Cardiac:** Symptomatic arrhythmia requiring interventional procedure: Pacemaker  **Cardiac:** Major cardiac medical procedure: Replacement  of ICD  **Cardiac:** Myocardial infarction |
| 20 | **Cardiac:** Symptomatic arrhythmia requiring interventional procedure: Defibrillator Implantation |  |
| 21 | **Cardiac:** Symptomatic arrhythmia interventional procedure: Reveal device implanted |  |
| 22 |  | **Cardiac:** Symptomatic arrhythmia requiring interventional procedure: Defibrillator Implantation |
| 23 |  | **Cardiac:** Symptomatic arrhythmia requiring direct current cardioversion and medication: Oral anticoagulant |
| 24 |  | **Cardiac:** Symptomatic arrhythmia requiring interventional procedure: Pacemaker |
| 25 |  | **Cardiac:** Major cardiac medical procedure: Heart transplant |
| 26 |  | **Cardiac:** Myocardial infarction |
| 27 |  | **2 Cardiac:** Congestive heart failure requiring hospitalisation or accompanied by elevated B-type natriuretic peptide |
| 28 |  | **Cardiac:** Symptomatic arrhythmia requiring interventional procedure: Pacemaker |
| 29 |  | **Death:** Cardiac |
| 30 |  | **Cardiac:** Symptomatic arrhythmia requiring interventional procedure: Defibrillator Implantation |

FACE, Fabry-associated clinical event; ICD, implantable cardioverter defibrillator; TIA, transient ischaemic attack.

## **Supplementary Table 2** List of serious adverse events.

| **Causality in relation to migalastat** | **Serious adverse event** |
| --- | --- |
| **Possibly related** | Transient ischaemic attack |
| **Unlikely to be related** | Atrial fibrillation |
|  | Myocardial infarction |
|  | Cardiac failure |
| **Unrelated** | 10 events relating to cardiac disorders |
|  | 1 event relating to renal and urinary disorders |
|  | 5 events relating to infection and infestations |
|  | 2 events relating to neoplasms benign, malignant and unspecified |
|  | 2 events relating to respiratory, thoracic and mediastinal disorders |
|  | 1 event relating to metabolism and nutrition disorders |
|  | 1 event relating to musculoskeletal and connective tissue disorders |
|  | 1 event relating to injury, poisoning and procedural complications |
|  | 1 event relating to nervous system disorders |
|  | 1 event relating to congenital, familial and genetic disorders |

## **Supplementary Table 3** Summary of amenable mutations by overall population and by sex.

| **Variant, *n* (%)** | **Overall (*N* = 125)** | **Males (*n* = 75)** | **Females (*n* = 50)** |
| --- | --- | --- | --- |
| p.N215S | 38 (30.4) | 32 (25.6) | 6 (4.8) |
| p.S238N | 10 (8.0) | 7 (5.6) | 3 (2.4) |
| p.F113L | 9 (7.2) | 9 (7.2) | 0 (0.0) |
| p.R301Q | 8 (6.4) | 5 (4.0) | 3 (2.4) |
| p.R301G | 6 (4.8) | 4 (3.2) | 2 (1.6) |
| p.R356W | 6 (4.8) | 2 (1.6) | 4 (3.2) |
| p.P205T | 5 (4.0) | 2 (1.6) | 3 (2.4) |
| p.A143T^a^ | 4 (3.2) | 1 (0.8) | 3 (2.4) |
| p.R118C^a^ | 3 (2.4) | 2 (1.6) | 1 (0.8) |
| p.I232T | 2 (1.6) | 1 (0.8) | 1 (0.8) |
| p.I317T | 2 (1.6) | 1 (0.8) | 1 (0.8) |
| p.M290T | 2 (1.6) | 1 (0.8) | 1 (0.8) |
| p.S345P | 2 (1.6) | 0 (0.0) | 2 (1.6) |
| p.T194I | 2 (1.6) | 0 (0.0) | 2 (1.6) |
| p.V254del | 2 (1.6) | 1 (0.8) | 1 (0.8) |
| p.A257P | 1 (0.8) | 0 (0.0) | 1 (0.8) |
| p.D165H | 1 (0.8) | 0 (0.0) | 1 (0.8) |
| p.E338K | 1 (0.8) | 0 (0.0) | 1 (0.8) |
| p.F337S | 1 (0.8) | 0 (0.0) | 1 (0.8) |
| p.G258R | 1 (0.8) | 0 (0.0) | 1 (0.8) |
| p.G271D | 1 (0.8) | 0 (0.0) | 1 (0.8) |
| p.G325S | 1 (0.8) | 0 (0.0) | 1 (0.8) |
| p.G35R | 1 (0.8) | 1 (0.8) | 0 (0.0) |
| p.G395A | 1 (0.8) | 0 (0.0) | 1 (0.8) |
| p.H46P | 1 (0.8) | 1 (0.8) | 0 (0.0) |
| p.I270T | 1 (0.8) | 0 (0.0) | 1 (0.8) |
| p.K240N | 1 (0.8) | 1 (0.8) | 0 (0.0) |
| p.M42T | 1 (0.8) | 0 (0.0) | 1 (0.8) |
| p.N139S | 1 (0.8) | 0 (0.0) | 1 (0.8) |
| p.N320H | 1 (0.8) | 1 (0.8) | 0 (0.0) |
| p.P205S | 1 (0.8) | 1 (0.8) | 0 (0.0) |
| p.P214L | 1 (0.8) | 1 (0.8) | 0 (0.0) |
| p.P259R | 1 (0.8) | 1 (0.8) | 0 (0.0) |
| p.P293T | 1 (0.8) | 0 (0.0) | 1 (0.8) |
| p.P362L | 1 (0.8) | 0 (0.0) | 1 (0.8) |
| p.R356G | 1 (0.8) | 0 (0.0) | 1 (0.8) |
| p.S126G | 1 (0.8) | 0 (0.0) | 1 (0.8) |
| p.W162G | 1 (0.8) | 0 (0.0) | 1 (0.8) |
| Missing^b^ | 1 (0.8) | 0 (0.0) | 1 (0.8) |

^a^Variants of uncertain significance.^1,2,3,4,5^
^b^Variant was not listed in the registry database but is known to be an amenable variant by the treating physician.

## **Supplementary Table 4** Patient demographics and disease characteristics at enrolment in the non-p.N215S group.

|  | **Non-p.N215S group** |
| --- | --- |
| *n* (%) | 87 (69.6) |
| **Age (years)** | |
| Median (range) | 56.0 (16.0–77.0) |
| >40 years, *n* (%) | 69 (79.3) |
| ≤40 years, *n* (%) | 18 (20.7) |
| **Sex (%)** | |
| Male | 43 (49.4) |
| Female | 44 (50.6) |
| **eGFR_CKD-EPI_, mL/min/1.73 m^2^** | |
| *n* (%) | 86 (98.9) |
| Mean (SD) | 85.5 (24.1) |
| eGFR ≥90 | 36 (41.4) |
| eGFR ≥60–90 | 37 (42.5) |
| eGFR ≥30–60 | 12 (13.8) |
| Missing | 1 (1.1) |
| **LVMi, g/m^2^** | |
| *n* (%) | 48 (55.2) |
| Median (range) | 115.3 (23.9–289.0) |

CKD-EPI, Chronic Kidney Disease Epidemiology Collaboration; eGFR, estimated glomerular filtration rate; LVMi, left ventricular mass index; SD, standard deviation.

## **Supplementary Table 5** Incidence of FACEs per 1000 patient-years in p.N215S patients and non-p.N215S patients.

| **FACEs, events per 1000 patient-years** | **p.N215S patients  (*n* = 38)** | **Non-p.N215S patients (*n* = 87)** |
| --- | --- | --- |
| Renal | 0 | 3.0 |
| Cardiac | 82.9^a^ | 83.4 |
| Cerebrovascular | 0 | 6.0 |
| Composite | 82.9 | 92.4 |

^a^Includes one death due to a cardiac event. FACE, Fabry-associated clinical event.

## **REFERENCES**

1. Ceron-Rodriguez M, Ramon-Garcia G, Barajas-Colon E, Franco-Alvarez I, Salgado-Loza JL. Renal globotriaosylceramide deposits for Fabry disease linked to uncertain pathogenicity gene variant c.352C>T/p.Arg118Cys: A family study. *Mol Genet Genomic Med.* 2019; 7:e981.

2. Ferreira S, Ortiz A, Germain GP, et al. The alpha-galactosidase A p.Arg118Cys variant does not cause a Fabry disease phenotype: data from individual patients and family studies. *Mol Genet Metab.* 2015; 114:248-258.

3. Lenders M, Weidemann F, Kurschat C, et al. Alpha-galactosidase A p.A143T, a non-Fabry disease-causing variant. *Orphanet J Rare Dis.* 2016; 11:54.

4. Spada M, Pagliardini S, Yauda M, et al. High incidence of later-onset fabry disease revealed by newborn screening. *Am J Hum Genet.* 2006; 79:31-40.

5. Valtola K, Nino-Quintero J, Hedman M, et al. Cardiomyopathy associated with the Ala143Thr variant of the α-galactosidase A gene. *Heart.* 2020; 106:609-615.
